# Extraction and purification of phycobiliproteins from algae and their applications

**DOI:** 10.3389/fchem.2022.1065355

**Published:** 2022-12-01

**Authors:** Gabriela Kovaleski, Mariam Kholany, Lília M. S. Dias, Sandra F. H. Correia, Rute A. S. Ferreira, João A. P. Coutinho, Sónia P. M. Ventura

**Affiliations:** ^1^ Department of Chemistry, CICECO—Aveiro Institute of Materials, University of Aveiro Campus Universitário de Santiago, Aveiro, Portugal; ^2^ Department of Physics, CICECO—Aveiro Institute of Materials, University of Aveiro Campus Universitário de Santiago, Aveiro, Portugal; ^3^ Instituto de Telecomunicações, University of Aveiro, Aveiro, Portugal

**Keywords:** phycobiliproteins, R-phycoerythrin, extraction, purification, applications

## Abstract

Microalgae, macroalgae and cyanobacteria are photosynthetic microorganisms, prokaryotic or eukaryotic, living in saline or freshwater environments. These have been recognized as valuable carbon sources, able to be used for food, feed, chemicals, and biopharmaceuticals. From the range of valuable compounds produced by these cells, some of the most interesting are the pigments, including chlorophylls, carotenoids, and phycobiliproteins. Phycobiliproteins are photosynthetic light-harvesting and water-soluble proteins. In this work, the downstream processes being applied to recover fluorescent proteins from marine and freshwater biomass are reviewed. The various types of biomasses, namely macroalgae, microalgae, and cyanobacteria, are highlighted and the solvents and techniques applied in the extraction and purification of the fluorescent proteins, as well as their main applications while being fluorescent/luminescent are discussed. In the end, a critical perspective on how the phycobiliproteins business may benefit from the development of cost-effective downstream processes and their integration with the final application demands, namely regarding their stability, will be provided.

## Introduction

Marine biomass is recognized worldwide as a valuable carbon source, which can be used for food, feed, chemicals, and biopharmaceuticals of paramount industrial relevance (Merlo et al., 2021). Algae are mostly known for their use in the production of biomaterials and biofuels, due to their high content of fats or polysaccharides (Pham et al., 2013). Nonetheless, new fields of application arise with a greater focus on the remaining compounds with multiple uses in the food, medical, pharmaceutical, and cosmetic industries. Both academia and industry have invested significant efforts during the last decades in the exploration of valuable bioproducts that can be sourced from algae, and which can allow the development of a biorefinery focusing on a blue economy. Plenty of high-value compounds such as proteins, antioxidants, vitamins, minerals, lipids, pigments, biopolymers (chitosan and sodium alginate), and polyunsaturated fatty acids are already being explored for this purpose (Barkia et al., 2019; Novak et al., 2019, Cuellar-Bermudez et al., 2014).

Microalgae, macroalgae, and cyanobacteria are photosynthetic microorganisms, prokaryotic or eukaryotic, living in saline or freshwater environments. The cell wall of macroalgae consists of polysaccharides (agar and cellulose), which are an obstacle to cell rupture during the extraction of their bioactive compounds (Mittal et al., 2017).

The species selection and cultivation strategies are considered essential to producing each compound of interest (López-Rodríguez et al., 2020), further boosting their industrial potential. Included in the set of bioactive compounds of most interests to academia and industry are the pigments, including chlorophylls, carotenoids, and phycobiliproteins (Pagels et al., 2019).

Phycobiliproteins are photosynthetic light-harvesting proteins present in cyanobacteria, red algae, cryptomonads, and cyanelles. They are water-soluble proteins, covalently bound *via* cysteine amino acid chromophores called phycobilins, which are open-chain tetrapyrroles (Mulders et al., 2014; Pagels et al., 2019), and organized in supramolecular structures called phycobilisomes, located in the stroma of the cells (Dumay et al., 2014).

The presence of phycobiliproteins in some organisms allows the transfer of light energy in spectral zones that cannot be used by chlorophyll a (responsible for the photosynthesis mechanism to occur), thus allowing the photosynthesis and the survival of living organisms even at low light intensities (Dumay et al., 2014) The phycobilisome works as an energetic funnel, allowing the energy transfer through chromophores to the reaction centers (Roy et al., 2011).

All phycobiliproteins have the same monomer as the basic unit, composed of α and β subunits. Each monomer can carry either one, two, or three chromophores, depending on the molecular species. These phycobilin chromophores are phycoerythrobilin (PEB), phycocyanobilin (PCB) and phycobiliviolin (PVB) (Bryant, 1982). Depending on the phycobiliprotein, different phycobilin combinations may occur leading to their specific spectral and optical identity (Glazer, 1994): Phycoerythrin with maximum absorption wavelengths (λmax) ranging between 490 and 570 nm (with three-peak absorption maxima at 565, 539, and 498 nm) (Liu et al., 2005); phycocyanin (λmax = 610–620 nm) (Dias et al., 2022) allophycocyanin (λmax = 650–655 nm), and phycoerythrocyanin (λmax = 560–600 nm) (Munier et al., 2014). Given that, phycobiliproteins differ in the amino-acid sequence, the number of chromophores per subunit, and the type of chromophores. Based on their structure and properties, specifically on their radiation absorption ability, phycobiliproteins are divided into four main types, namely phycoerythrin (PE), phycocyanin, phycoerythrocyanin, and allophycocyanin, as detailed in Table 1 (Pagels et al., 2019).

**TABLE 1:** Main properties and characteristics of phycobiliproteins.

Phycobiliprotein	Absorption maxima (nm) (Liu et al., 2005)	Chemical structure (Li et al., 2019)	Molecular weight (kDa)	Chromophore ID and structure
Phycoerythrin	490–570	(αβ)6γ complexes	240	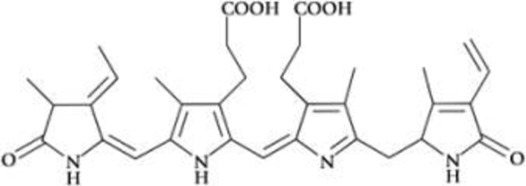 Phycoerythrobilin (PEB)
Phycoerythrocyanin	560–600	(αβ)_3_		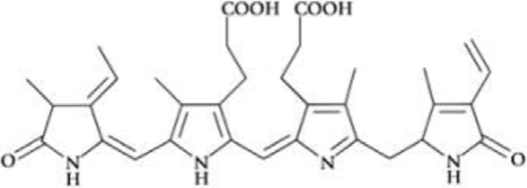 Phycoerythrobilin (PEB)
Phycocyanin	610–625	(αβ)_3_	30	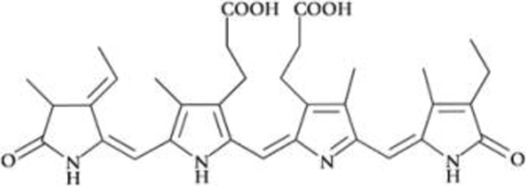 Phycocyanobilin (PCB)
Allophycocyanin	650–660	(αβ)_3_	104	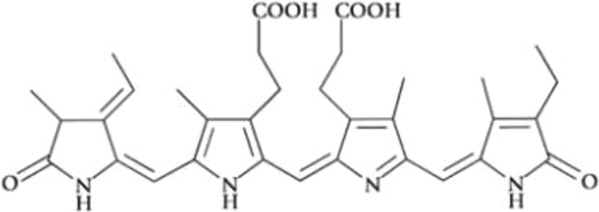 PCB and 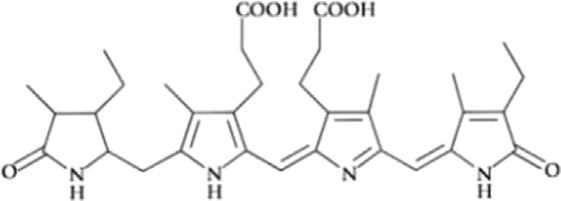 Phycobiliviolin (PVB)

PE is found in the chloroplasts of red algae, cyanobacteria, being generally composed of (αβ) 6γ complexes (α, 18–20 kDa; β, 19.5–21 kDa; and γ, 30 kDa) (Munier et al., 2014), with a total molecular weight around 240 kDa (Table 1). PE can be classified into four classes: B-PE (Bangiophyceae PE, containing PEB only or containing PEB and phycourobilin) C-PE (cyanobacterial-PE), and R-PE (Rhodophyta-PE). The increment of the γ subunit in R-PE in comparison with other phycobiliproteins confers additional stability since this subunit is in the center of the molecule linking the (αβ) 3 trimers (Wang et al., 1998). Indeed, R-PE is recognized for its stability towards several denaturant agents, namely temperature and pH (Galland-Irmouli et al., 2000). The high solubility in water and stability associated with R-PE has increased industrial interest. R-PE is commonly used as a natural colouring agent (Kamble et al., 2018), fluorescent label probe (Wang et al., 2020), and as an ingredient in pharmaceutical formulations (Sekar and Chandramohan, 2007). Many studies show the various biological activities of R-PE, namely its antioxidant and anti-cancer properties (Pan et al., 2013; Jung et al., 2016; Tan et al., 2016).

Given the broad range of applications of phycobiliproteins, particularly of R-PE, and their consequent economic value, there is a growing interest in the development of more sustainable and efficient extraction and purification techniques for their recovery. These methods are dependent on the biomass and should be tailored in accordance (Ranjitha and Kaushik, 2005). Cell disruption, primary recovery, and purification are the three main steps for the recovery of pure R-PE. The polysaccharides present in the algal cell wall, such as agar and cellulose, interfere with cell disruption during extractions, so there is a need for a suitable method for PE extraction (Mittal et al., 2017). The purity index expressed as the A_565_ nm/A_280_ nm ratio indicates the purity of PE for different applications, where a value of 0.7 represents a protein with food-grade purity, 3.9 as a reactive grade, and greater than 4.0 as analytical grade (Rito-Palomares et al., 2001). Some other variables to be considered are the recovery, the yield of extraction, the extraction efficiency, and the purity level, which will be further described and analysed in this work.

This review will focus on the different downstream schemes reported so far for the recovery of phycobiliproteins from marine and freshwater biomass, highlighting the different sources, namely macroalgae, microalgae, and cyanobacteria, the solvents and techniques applied in the extraction and purification of the fluorescent proteins, as well as their main applications while being fluorescent/luminescent. In the end, a critical perspective on how the sector of phycobiliproteins may benefit from the development of cost-effective downstream processes and their integration with the final application demands, namely regarding their stability, will be provided.

## Downstream processing

A downstream process is traditionally defined by two or three main steps, which depend on the compounds to be recovered from the biomass being produced extra or intracellularly. Moreover, the downstream processes to apply, depends not only on the type of biomolecule(s) to recover (considering the physical, chemical, and optical properties) but also on the morphology of the raw material, in the specific case of this review, cyanobacteria, macro or microalgae.

Considering that the focus of this review is the phycobiliproteins, the downstream processes associated with their production are composed of three main steps: 1) cell disruption and pigments’ release, 2) extraction of phycobiliproteins 3) purification of phycobiliproteins by separating them from the other contaminants. As recently discussed by Martins and Ventura (2020), the traditional schemes of cell disruption and biomolecules release are based on mechanical and/or chemical treatments. Included in the mechanical treatments, and considering specifically the release of phycobiliproteins, the effect of maceration, freeze-thaw, ultrasounds, and microwaves have been evaluated. In addition to the mechanical treatments, two other techniques are explored to recover phycobiliproteins, namely the use of specific solvents in the solid-liquid extraction of the pigments and the enzymatic hydrolysis (as described in Tables 2, 3). Furthermore, with a much lower incidence of investigation are the extraction schemes considering the combination of mechanical and chemical treatments (Martínez et al., 2019). Normally, the first step for the recovery of a valuable compound produced intracellularly by any organism is cell disruption with the consequent release of the cell components (Günerken et al., 2015). Disruption processes have been effectively performed to release R-PE from algae by applying the techniques of maceration, freeze-thaw, ultrasound, microwave irradiation and enzymatic hydrolysis. Normally these techniques are used in conjunction with solid-liquid extraction, which can be water or other solvents. In the next section of this review, a brief analysis of the techniques applied to extract phycobiliproteins from 1) macroalgae and 2) microalgae/cyanobacteria.

**TABLE 2:** Extraction methods applied so far to the recovery of phycobiliproteins from macroalgae.

Species	Tissue disruption/Extraction method	Yield/Extraction efficiency PE	Yield/Extraction efficiency PC	Yield/Extraction efficiency	R-PE purity index (A_565_/A_280_)	References
*Gracilaria gracilis*	Maceration (mortar and pestle)	3.58 ± 0.03 mg g^−1^	0.62 ± 0.02 mg g^−1^	—	—	Pereira et al. (2020)
	Ultrasonic bath	1.60 ± 0.12 mg g^−1^	0.37 ± 0.03 mg g^−1^	—	—	
	Ultrasonic probe	1.57 ± 0.10 mg g^−1^	0.44 ± 0.01 mg g^−1^	—	—	
	High pressure	0.25 ± 1.27 mg g^−1^	—	—	—	
	Freeze-thawing	1.51 ± 0.03 mg g^−1^	—	—	—	
	Maceration with pestle and mortar	7 mg g^−1^ d.w.	2 mg g^−1^ d.w.	—	—	Francavilla et al. (2015)
	Aqueous solutions of ionic liquids	0.4 mg g^−1^ fresh biomass	-	—	—	Martins et al. (2016)
*Gelidium pusillum*	Ultrasonication	0.16 ± 0.01 mg g^−1^	0.11 ± 0.01 mg g^−1^	—	—	Mittal et al. (2017)
	Maceration using mortar and pestle	1.19 ± 0.03 mg g^−1^	0.81 ± 0.03 mg g^−1^	—	—	
	Maceration in liquid nitrogen	0.54 ± 0.05 mg g^−1^	0.34 ± 0.03 mg g^−1^	—	—	
	Homogenization	1.29 ± 0.04 mg g^−1^	0.80 ± 0.07 mg g^−1^	—	—	
	Freezing-thawing	0.17 ± 0.04 mg g^−1^	0.29 ± 0.02 mg g^−1^	—	—	
	Maceration + freezing-thawing	0.9 ± 0.03 mg g^−1^	0.61 ± 0.02 mg g^−1^	—	—	
	Homogenization + ultrasonication.	1.41 ± 0.01 mg g^−1^	0.95 ± 0.01 mg g^−1^	—	—	
	Maceration + ultrasonication	1.56 ± 0.01 mg g^−1^	1.19 ± 0.01 mg g^−1^	—	—	
*Grateloupia turuturu*	Ultrasound-assisted	—	—	—	—	Guillard et al. (2015)
	Ultrasound-assisted extraction + enzymatic hydrolysis	3.6 mg g^−1^ (22°C)	—	—	—	
*Porphyridium purpureum*	Microwave-Assisted (40°C)	73.7 ± 2.3 μg mg^−1^	34.8 ± 6.4 μg mg^−1^	—	—	Juin et al. (2014)
*Porphyridium cruentum*	Fresh: Freeze-thawing (−20°C and 20–25°C)	71 ± 4%	—	—	—	Lauceri et al. (2019)
	Fresh: Freeze-thawing + Ultrasound	69 ± 3%	—			
	Freeze dried: Freeze-thawing (−20°C and 20–25°C)	69 ± 5%	—			
	Freeze dried: Freeze-thawing + Ultrasound	62%	—			
	50 mM acetate buffer at pH 5.5) + five repeated freeze-thaw cycles	0.27 mg ml^−1^	—	—	—	Ibáñez-González et al. (2016)
*Pyropia yezoensis*	Freeze-thaw (−20°C and 4°C)	—	—	3.766 ± 0.021 mg g^−1^ dw	0.195 ± 0.015	Wang et al. (2020)
	Maceration	—	—	2.465 ± 0.017 mg g^−1^ dw	0.176 ± 0.014	
	Hydrolysis	—	—	2.087 ± 0.022 mg g^−1^ dw	0.147 ± 0.012	
	Enzymatic hydrolysis (agarase and cellulase)	—	—	6.953 ± 0.020 mg g^−1^ dw	0.287 ± 0.014	
*Mastocarpus stellatus*	Enzymatic hydrolysis (xylanase)	—	—	1.99 mg g^−1^ dw	0.36	Nguyen et al. (2016)
*Palmaria palmata*	Enzymatic digestion (xylanase)	—	—	3.28 ± 0.64 (g.kg^−1^ dw)	0.14 ± 0.03	Dumay et al. (2013)
	After optimization	—	—	12.36 ± 0.37 (g.kg^−1^ dw)	0.40 ± 0.04	
*Gracilaria verrucosa*	Enzymatic hydrolysis (endocellulase and βxylanase)	—	—	6.25 mg g^−1^	-	Mensi et al. (2011)

d.w. (dry weigh).

[PE], phycoerythrin concentration; [PC], phycocyanin concentration.

**TABLE 3:** Extraction methods applied so far to the recovery of phycobiliproteins from microalgae and cyanobacteria.

Species	Tissue disruption/Extraction method	Yield/Extraction efficiency PE	Yield/Extraction efficiency PC	References
*Spirulina maxima*	Ultrasonication	0.8 mg ml^−1^	11.3 mg ml^−1^	Choi and Lee (2018)
*Spirulina platensis*	Ultrasonication + protic ionic liquids (2-HEAA + 2-HEAF)	—	0.75 g.L^−1^	Rodrigues et al. (2018)
	Mechanical agitation + thermal heating + protic ionic liquids (2-HEAA + 2-HEAF)	—	1.65 g.L^−1^	Rodrigues et al. (2019)
*Pseudanabaena catenate*	Three cycles of repeated freezing in liquid nitrogen + maceration mortar and pestle.	25.5 ± 5.1 mg.L^−1^	28.8 ± 2.8 mg.L^−1^	Khan et al. (2018)
*Pseudanabaena amphigranulata*	Three cycles of repeated freezing in liquid nitrogen + maceration mortar and pestle.	10.2 ± 3.9 mg.L^−1^	86 ± 14.7 mg.L^−1^	Khan et al. (2018)
*Arthrospira platensis GL*	Fresh: Freeze-thawing (−20°C and 20–25°C)	—	77 ± 6%	Lauceri et al. (2019)
	Fresh: Freeze-thawing + Ultrasound	—	76 ± 6%	
	Freeze dried: Freeze-thawing (−20°C and 20–25°C)	—	81 ± 2%	
	Freeze dried: Freeze-thawing + Ultrasound	—	79 ± 1%	
*Porphyridium cruentum*	50 mM acetate buffer at pH 5.5) + five repeated freeze-thaw cycles	0.27 mg ml^−1^	—	Ibáñez-González et al. (2016)
	Fresh: Freeze-thawing (−20°C and 20–25°C)	71 ± 4%	—	Lauceri et al. (2019)
	Fresh: Freeze-thawing + Ultrasound	69 ± 3%	—	
	Freeze dried: Freeze-thawing (−20°C and 20–25°C)	69 ± 5%	—	
	Freeze dried: Freeze-thawing + Ultrasound	62%	—	
*Porphyridium purpureum*	Microwave-Assisted (40°C)	73.7 ± 2.3 μg mg^−1^	34.8 ± 6.4 μg mg^−1^	Juin et al. (2014)

## Extraction processes applied to macroalgae

### Conventional techniques

Macroalgae, also known as seaweeds, are multicellular, macroscopic algae, which may belong to different groups of multicellular algae: green, red, and brown algae (Suganya et al., 2016). Due to their desirable characteristics, such as high photosynthetic efficiency, high biomass conversion rate, ease of handling, and fast growth rate, they are considered a promising raw material for biotechnological valorization answering the needs of a marine biorefinery (Francavilla et al., 2015). The cell wall of macroalgae consists of polysaccharides (agar and cellulose), which are an obstacle to cell rupture during the extraction of their bioactive compounds (Mittal et al., 2017). Maceration and milling are often used, with liquid nitrogen freezing to yield better results. However, at least some of these cell disruption approaches require increased time, specific equipment, and higher overall costs. An example is the use of liquid nitrogen at a lab scale which is impossible to apply in higher scales, but nevertheless, it can be replaced by a cryogenic mill operational unit. Ultrasonication is a technique where biomass breaks down by the compression and decompression cycles resulting from sound waves at frequencies normally higher than 20 kHz, also requires less time and lower temperature (Guillard et al., 2015; Mittal et al., 2017). Table 2 describes the yields of extraction and purities obtained by the application of different conventional methods.

In 2015, Francavilla et al. (2015) used maceration to extract phycobiliproteins from *Gracilaria gracilis*, which was used as the first step of a complex biorefinery cascade, achieving a yield of 7 mg PE. g^−1^ d. w. and 2 PC. g^−1^ d. w. Later, Pereira and co-authors (2018) compared five techniques for the extraction of R-PE from the same algae, namely maceration, ultrasonic bath, ultrasonic probe, high pressure, and freeze-thawing. Using a Response Surface Methodology for optimization of the extraction method, a greater efficiency was attained through maceration with mortar and pestle yielding an extraction of 3.58 ± 0.03 mg PE. g^−1^ and 0.62 ± 0.02 mg PC. g^−1^, confirming that PE is the most abundant phycobiliprotein in *Gracilaria gracilis*. Still, in the study of red macroalgae, various extraction methods were tested on *Gelidium pusillum*, namely maceration with freezing-thawing, homogenization and ultrasonication, and maceration and ultrasonication, the latter being more effective in the R-PE and R-PC extraction, 77%, and 93%, respectively (Mittal et al., 2019). Guillard et al. (2015) compared two extraction processes with *Grateloupia turuturu*, ultrasound-assisted extraction and ultrasound-assisted with enzymatic hydrolysis. Despite the higher complexity of an enzymatic step, normally a better performance is achieved considering the specificity of the enzymes to break the bonds between the constituents of the biomass (3.6 mg g^−1^ at 22°C). Finally, in 2017, Sharmila et al (2017) used different cell disruption schemes, which included the maceration using mortar and pestle, the freeze-thaw, the use of lysozyme and sonication for the extraction of phycobiliproteins from *Kappaphycus alvarezii*. In this work, the authors have also investigated different process conditions, namely, three temperatures for the freeze-thaw, the best extraction using freeze-thaw at a temperature of −20°C–25°C.

### Solvent-and solvent-assisted extraction

Another approach for the extraction of molecules is the use of solvents. Phycobiliproteins are hydrophilic proteins, thus, conventional solvents used in their extraction are mainly water or buffers (to control the media pH). These solutions can be phosphate buffer, ethylenediamine tetra-acetic acid (EDTA), acetate buffer, or even water. For the optimization of the extraction, Hemlata et al. (2018) have used five different buffers as solvents to extract phycoerythrin from Michrochaete, namely the citrate buffer (pH-5.0; 0.1 M), acetate buffer (pH-6.0; 0.1 M), carbonate buffer (pH 9.6; 0.1 M), Tris-HCl buffer (pH-7.2; 0.05 M) and the sodium phosphate buffer (pH-7.0; 0.1 M). After optimization, a higher yield of extraction (65.21 mg g^−1^) was obtained with the acetate buffer (pH-6.0; 0.1 M). They also showed the antioxidant, antibacterial, anticancer, antifungal activities of Microchaete’s PE. Sfriso et al. (2018) used different concentrations of buffers, phosphate buffer (0.1, 1, 10, and 100 mM), and EDTA (0.1, 1, 10, and 100 mM), to later investigate the fluorescence of PE. Sharmila et al. (2017) also optimized the process with different buffers at different pH conditions, followed by different cell disruption methods, and this result was found for different temperatures. The results were better with sodium phosphate pH 7.2 and using freeze-thaw at −20°C/−25°C. Sintra and co-authors (2021) also used sodium phosphate for extraction and achieved 90% of recovery of C-PC.

Meanwhile, Nguyen et al. (2016) compared different concentrations of phosphate buffer (20 mM, 50 mM, and 0.1 M) with tap and pure water with maceration in liquid nitrogen. It was found that the solution of phosphate buffer 20 mM with pH 7.1 showed better results for PE in *Mastocarpus stellatus*. Sudhakar et al. (2015) also purchased the extractions of the algae *Gracilaria crassa* with water (distilled water and seawater) and phosphate buffer (0.1 M), and found a better yield for distilled water for PE (0.35 mg g^−1^) and PC (0.18 mg g^−1^). The use of solvents was also reported by its combination with microwave irradiation. Microwave irradiation consists of instantaneous and homogeneous heat transfer in the sample to break the cell wall. Juin et al. (2014) achieved maximum extraction efficiency of PE (73.7 ± 2.3 μg mg^−1^) with just 10 s of irradiation, at 40°C, showing that this procedure is fast and has high yields, but for PC the efficiency was lower (34.8 ± 6.4 μg mg^−1^) with 10 s but with a temperature of 100°C, describing that: “The weak extractability of the two pigments tightly bound to the thylakoid membrane compared to PE.” Martins et al., 2016 compared the extraction of PBPs in *Gracilaria* sp. between sodium phosphate and different ionic liquids, finding cholinium chloride as the best solvent, with an increase of 45% in yield and represented by high selectivity since practically no chlorophylls were extracted simultaneously. Pressurized liquids extraction (PLE), which is a method that uses solvents at high temperatures and pressures for the extraction of compounds, has the advantage of being a faster process and using less solvent. This method was applied in the extraction of PE and proved to be efficient when the temperatures were lower and with pressurized water (16.51 ± 0.21 mg g^−1^ of PE) (Gallego et al., 2019).

## Extraction processes applied to microalgae and cyanobacteria

### Conventional techniques

Cyanobacteria are unique photosynthetic organisms present in almost all habitats all over the world, as pointed out by the World Health Organization (WHO, 2021). They have a small cell size and can be unicellular, filamentous, or colonial, being sometimes large enough to be visible by the human eye, especially during the occurrence of natural blooms (Macário et al., 2021). These bacteria have been studied for their morphology, photosynthesis, and nitrogen fixation mechanisms, but also for certain aspects of their structure namely in what concerns the part of the cell driving photosynthesis. As recurrently reported, the cyanobacteria photosynthetic apparatus is composed of three light-harvesting systems, namely the two main photosystems found in other photosynthetic organisms and a phycobilisome (Masojídek et al., 2013). The phycobilisome of these organisms is mainly composed of phycobiliproteins, the phycobilisome composition varying from species to species.

Microalgae are microscopic algae, unicellular, which may vary in size from a few micrometers to a few hundred of micrometers (Suganya et al., 2016). They can produce hydrogen, hydrocarbons, fats and carbohydrates, as well as be able to use different water sources, such as fresh, saline, and wastewater (Randrianarison and Ashraf, 2017). Most microalgae/cyanobacteria produce more phycobiliproteins under stressful environmental conditions, especially light (Manirafasha et al., 2016). Microalgae have already been incorporated, with good acceptability, in dairy products as bioactive compounds (Caporgno and Mathys, 2018).

Although most works report the recovery of phycobiliproteins from macroalgae, Choi and Lee (2018) have extracted phycobiliproteins from *Spirulina* sp. (a cyanobacterium commonly used as a functional food) with ultrasound and obtained very high amounts of phycocyanin (11.3 mg ml^−1^) when compared to conventional water extraction at 4°C (9.8 mg ml^−1^) and 25°C (5.7 mg ml^−1^). For PE a low yield of 0.8 mg ml^−1^ was obtained, demonstrating that PE is not an abundant phycobiliprotein in this species. In the same year, Khan et al. (2018) studied the production of PC and PE in two different strains of *Pseudanabaena*. *P. catenata* produced more PE in green light (25.5 ± 5.1 mg.L^−1^) but *P. amphigranulata* produced 86 ± 15 mg.L^−1^ of PC in red light. For that, the authors have used three cycles of freezing-thawing of biomass in liquid nitrogen and then maceration using a mortar and pestle.

Included in the criteria to select the species to explore in the recovery of phycobiliproteins should also be the need for a pre-treatment of the cells before cell disruption. Following this rationale, the difference between the use of fresh or freeze-dried biomass was evaluated with the freeze-thawing and freeze-thawing + ultrasound process by Lauceri et al. (2019). For *Arthrospira platensis GL* the yield of PC was 81% for the frozen microalgae in freeze-thawing extraction, whereas in *Porphyridium cruentum* the higher recovery yield with the fresh algae (71%) was obtained for PE, which was independent of the method of extraction employed. Another study with fresh *Porphyridium cruentum*, using five repeated freeze-thaw fresh cycles was carried reporting a higher recovery yield of 86.6%, this value representing a concentration of 0.27 mg ml^−1^ of R-PE (Ibáñez-González et al., 2016).

### Solvent and solvent-assisted extraction

For the optimization of the extraction, Hemlata et al. (2018) have used five different buffers as solvents to extract phycoerythrin from Michrochaete, namely the citrate (pH-5.0; 0.1 M), acetate (pH-6.0; 0.1 M), carbonate (pH9.6; 0.1 M), Tris-HCl (pH-7.2; 0.05 M) and the sodium phosphate buffers (pH-7.0; 0.1 M). After optimization, a higher yield of extraction (65.21 mg g^−1^) was obtained with the acetate buffer (pH-6.0; 0.1 M). They also showed the antioxidant, antibacterial, anticancer, and antifungal activities of Microchaete’s PE. Sfriso et al. (2018) used different concentrations of buffers, phosphate buffer (0.1, 1, 10, and 100 mM), and EDTA (0.1, 1, 10, and 100 mM), to later investigate the fluorescence of PE. Sharmila et al. (2017) also optimized the process with different buffers at different pH conditions, followed by different cell disruption methods and this result was found for different temperatures. The results were better with sodium phosphate pH 7.2 and using freeze-thaw at -20°C/-25°C. Sintra et al. (2021) also used sodium phosphate for extraction and achieved 90% of recovery of C-PC.

The use of protic ionic liquids (PIL) was also studied since the operating conditions required are softer compared to other alternatives. As ILs are expensive, PILs were investigated for their lower price. Rodrigues et al. (2018) used the PILs on *Spirulina (Arthrospira) platensis* in combination with ultrasonic and obtained a PC concentration of 0.75 g.L^−1^ with PIL 2-HEAA + 2-HEAF. In 2019, Rodrigues et al. (2019) were able to double the concentration (PC concentration of 1.65 g.L^−1^) when PILs were used with mechanical agitation and thermal heating in *Spirulina platensis* and with the same PIL (2-HEAA + 2-HEAF).

At this point, and considering the works reviewed, it is not completely clear what should be considered the most appropriate technique to extract the phycobiliproteins from the different algal matrices. However, it is clear from the data that techniques like a microwave- and ultrasound-assisted extractions, as well as the use of only buffers as solvents, although less expensive, do not allow the development of processes of extraction with high selectivity. Nevertheless, it seems that *Spirulina* species is one of the simplest to process since the yields of extraction are higher than the ones obtained for the remaining species analysed. Moreover, the comparison between the results presented in Table 3 seems to suggest that ultrasonication combined with the use of ionic liquids is the best approach to extracting phycocyanin. Nevertheless, it should also be pointed out that the number of works is not so significant to allow us to define some heuristic rules on the best mechanical approaches or even on the best solvents to apply. One point is, however, clear; ionic liquids are normally recognized as being more selective solvents (Martins et al., 2016), although the selectivity was not checked in the works analysed.

### Purification

Depending on the final application envisioned for PE, namely in the energy, food, cosmetic, or pharmaceutical industries, different purities are required, which greatly affect the production cost and the product price (Torres-Acosta et al., 2016). Regardless of its efficiency, the extraction process often lacks selectivity. Low selectivity means the low purity of the extracts obtained. Solutions of purified phycobiliproteins are expensive, considering the established markets (e.g., as natural food colorants), but also new market applications with high economic and industrial relevance (energy, medical, pharmaceutical, and cosmetic). For reference, a purity index, expressed as the A565 nm/A280 nm ratio, of 0.7 represents a protein with food-grade purity, 3.9 as a reactive grade, and greater than 4.0 as analytical grade (Rito-Palomares et al., 2001).

### Chromatographic techniques

The most extensively used purification technique is chromatography, which can be ion-exchange, expanded-bed absorption, or reverse-phase (Table 4). Often, the purification consists of a combination of techniques to reach higher purity levels. A typical example is the use of precipitation followed by chromatography. Nguyen et al. (2019) achieved a high purity index (3.3) of R-PE from *Gracilaria gracilis* after purification on DEAE-Sepharose fast flow chromatography. The use of ammonium sulfate before chromatography is very common since it can remove amino acids, and consequently increase the purity of PE (Lee et al., 2017). Gargouch et al. (2018) used two-step precipitation with ammonium sulfate (first 20% and second 40%) before extraction on DEAE-Cellulose in the *Porphyridium marinum* algae and achieved a high PE purity (5.0). Senthilkumar et al. (2013) used only precipitation by ammonium sulfate (55%), obtaining a high PE purity (5.2) from red alga *Portieria hornemannii*. The use of ultrafiltration before anion exchange chromatography (SOURCE 15Q) was evaluated in the microalgae *Porphyridium cruentum*, achieving an analytical grade B-PE at the commercial level (purity index of 5.1). Munier et al. (2015) studied the difference between using only ammonium sulfate precipitation for PE purification and in combination with anion-exchange chromatography (DEAE-Cellulose), with the purity index increasing from 1.2 to 2.9.

**TABLE 4:** Purification methods applied to the fractionation of phycobiliproteins.

Type of algae	Species	Tissue disruption/Extraction method	Purification method	Yield/Extraction efficiency/Recovery/PE purity index	Yield/Extraction efficiency/Recovery/PC purity index	References
Macroalgae	Gracilaria gracilis	Phosphate buffer 20 mM	Anion-exchange chromatography (DEAE Sepharose)	0.24 ± 0.01 mg g^−1^ A_565_/A_280_ = 3.25 ± 0.01	—	Nguyen et al. (2019)
		Maceration	Induced precipitation + ultrafiltration (Poly (acrylic acid) sodium salts)	79.5% yield	—	Martins et al. (2021)
	Pyropia haitanensis residue	Freeze-thaw	Expanded-bed chromatographic (DEAE-Sepharose.)	[PE] 247.13 mg.L^−1^ OD_565_/OD_280_ = 4.01		Zhao et al. (2019)
	Halymenia floresia	0.05 M phosphate buffer at pH 7.0	Polyacrylamide Gel Using Electrophoretic elution technique (Preparative Native PAGE + dialyzed	41.1% yield A_565_/A_280_ = 5.9	—	MalairajMuthu et al. (2016)
	Grateloupia turuturu	Liquid nitrogen + sodium phosphate buffer (20 mM; pH 7.1)	Ammonium sulfate precipitation 85%	A_565_/A_280_ = 1.22	—	Munier et al. (2015)
			+ Anion-exchange chromatography (DEAE-Cellulose)	A_565_/A_280_ = 2.89		
	Porphyra yezoensis Ueda	Phosphate buffered saline + EDTA	Continuous precipitation with ammonium sulfate at different concentrations (10%, 20%, 40% and 50%) + Hydroxylapatites chromatography (HAC)	A_565_/A_280_ = 5.50	A_615_/A_280_ = 5.10	Cai et al. (2014)
	Gracilaria corticata	Phosphate buffer (0.1 M)	65% ammonium sulphate + dialyzed	0.24 mg g^−1^	0.11 mg g^−1^	Sudhakar et al. (2014)
			+ Anion-exchange chromatography (DEAE-Cellulose)	A_565_/A_280_ = 1.10	—	
	Portieria hornemannii	0.02 mM phosphate buffer at pH 7.2 + freezing-thawing	Ammonium sulfate (55%) + anion exchange column chromatography (Q-Sepharose)	A_562_/A_280_ = 5.2	—	Senthilkumar et al. (2013)
	Gracilaria lemaneiformis	10 m*M* phosphate buffer (*p*H 6.8) + agar + freeze-thaw	Anion-exchange chromatography (DEAE-Sepharose)	Recovery 16%	OD_565_/OD_280=3.2_	Niu et al. (2013)
	Porphyra yezoensis	10 mM phosphate buffer (pH 6.8) + freeze–thaw	Expanded bed chromatography (Phenyl-sepharose)	0.96 mg g^−1^	OD_565_/OD_280_ = 2.0–2.5	Niu et al. (2010)
			Anion-exchange chromatography (DEAE-Sepharose)	0.82 mg g^−1^	OD_565_/OD_280_ = 4.5	
	Corallina elongata	10 mM sodium phosphate Ph7+filtration	Hydroxyapatite chromatography	A_566_/A_280_ = 6.67	—	Rossano et al. (2003)
	Ceramium isogonum	1 mM K-phosphate (pH 6.8)	Ion-exchange chromatography (DEAE)	A_565_/A_280_ = 2.10	—	Kaixian et al. (1993)
Microalgae or cyanobacteria	Porphyridium marinum	Sodium phosphate buffer (20 mM, pH = 7.2) + freezing-freezing + ultrasound	Two steps of precipitation with ammonium sulfate + Dialyzed + anion exchange chromatography (DEAE-Cellulose)	57 mg g^−1^ dry weight	—	Gargouch et al. (2018)
				Recovery = 72% A_545_/A_280_ = 5		
	Bangia atropurpurea	50 mM phosphate buffer (pH 7.2) + sonicated	35% saturated ammonium sulfate + dialyzed	64.8% recovery A_562_/A_280_ = 2.47	54.7% recovery A_615_/A_280_ = 0.77	Punampalam et al. (2018)
			Gel filtration with Sephadex G-200	91.3% recovery A_562_/A_280_ = 4.76	68.3% recovery A_615_/A_280_ = 2.80	
			Reverse Phase-High Performance Liquid Chromatography (RP-HPLC)	100% recovery A_562_/A_280_ = 5.42	100% recovery A_615_/A_280_ = 3.95	
	Nostoc sp. strain HKAR-2	50 mM potassium phosphate buffer (pH 7.0) + sonication + repeated freezing	Ammonium sulfate precipitation (20–70%) + Dialyzed + Gel filtration chromatography (Sephacryl S-100 HR)	A_563_/A_280_ = 7.2	A_615_/A_280_ = 3.18	Kannaujiya and Sinha (2016b)
	Nostoc sp. strain HKAR-11	50 mM phosphate buffer (PB) (pH 7.0)	Ammonium sulfate precipitation	97% recovery A_563_/A_280_ = 1.10	96% recovery A_615_/A_280_ = 0.92	Kannaujiya and Sinha (2016a)
		+ mortar and pestle + repeated freeze	+ Gel filtration chromatography (Sephacryl S-100 HR)	89%, recovery A_563_/A_280_ = 6.37	80% recovery A_615_/A_280_ = 1.36	
			+ Hydrophobic interaction chromatography	83% recovery A_563_/A_280_ = 11.53	73% recovery A_615_/A_280_ = 5.75	
	Porphyra yezoensis	10 mM phosphate buffer (pH 6.8) + freeze–thaw	Expanded bed chromatography (Phenyl-sepharose)	0.96 mg g^−1^	OD_565_/OD_280_ = 2.0–2.5	Niu et al. (2010)
			Anion-exchange chromatography (DEAE-Sepharose)	0.82 mg g^−1^	OD_565_/OD_280_ = 4.5	

Sudhakar et al. (2014) purified the PE from red seaweed *Gracilaria corticate* found abundantly in Indian waters throughout the seasons, through anion-exchange chromatography, to study the stability in carbonated drinks as a natural coloring, concluding that PE can be used in cool, sweetened, and carbonated drinks. The use of gel filtration (Sephacryl S-300) before anion-exchange chromatography was found for the extraction of PE in *Lyngbya arboricola* and *Synechococcus sp*, with a purity index of A_560_/A_280_ = 5.2 and A_542_/A_280_ = 3.4, respectively (Tripathi et al., 2007; Kim et al., 2010).

Expanded bed adsorption chromatography is a suitable technique for protein recovery without the need for prior clarification. Bermejo et al. (2007) used this technique with *Porphyridium cruentum* achieving 66% of PE recovered. Niu et al. (2010) compared the expanded bed and anion-exchange chromatography in *Porphyra yezoensis*, the largest and most important aquaculture species in China, achieving a higher yield from expanded bed adsorption but a higher purity ratio in anion-exchange chromatography. The use of this technique was efficient for the purification of PE in *Pyropia haitanensis* residue, with a concentration of 247.13 mg.L^−1^ and purity index of 4.1 (Zhao et al., 2019).

Rossano et al. (2003) used hydroxyapatite for the purification of PE, which is a chromatographic resin that can be produced at a very low cost, achieving an optimal purity index of 6.7. Another study on *Porphyra yezoensis* Ueda used chromatography with hydroxyapatite as adsorbent material after continuous precipitation with ammonium sulfate and obtained a purity ratio of 5.5 of PE and 5.1 of PC (Cai et al., 2014).

The cyanobacterium *Nostoc sp.* has proved to be an excellent source of PE. Kannaujiya and Sinha, (2016) performed the purification with ammonium sulfate precipitation and gel filtration chromatography (Sephacryl S-100 HR) obtaining a high purity of PE (7.2). In another study, another purifying process was added, namely a hydrophobic interaction chromatography, allowing to obtain a purity of 11.5. Punampalam et al. (2018) extracted phycobiliproteins with saturated ammonium and isolated PE and PC by gel filtration (Sephadex G-200) and further purified by Reverse Phase-High Performance Liquid Chromatography (RP-HPLC), demonstrating a higher extraction and purity ratio for PE, while the protein had its antioxidant activity improved. MalairajMuthu et al. (2016) obtained the optimum purity of 5.9 from *Halymenia floresia* using an alternative to chromatography, the electrophoretic elution technique. Another purification used as ultrafiltration. Marcati et al. (2014) used ultrafiltration to separate PE from high molecular weight polysaccharides in *Porphyridium cruentum*, first using a 300,000 Da membrane and then a second with 10,000, leaving PE with a purity index of 2.3. Finally, in 2021, Martins et al. (2021) found that precipitation with ammonium sulfate has a good yield for R-PE and R-PC (100% and 81.1%, respectively), however, it was not selective for any of the PBPs, unlike using poly (acrylic acid) sodium salts as precipitation agents and conjugated with an ultrafiltration step (in this case R-PE was precipitated after extraction from *Gracilaria gracilis*, with a yield of 79.5%.

### Aqueous biphasic systems

ABS consists of a liquid-liquid extraction, where the biphasic system can be achieved by mixing two hydrophilic and non-miscible polymers or one salt and one polymer. Table 5 summarizes the conditions, yields of extraction and purities attained for the extraction of PE and PC using ABS in the various reports on the subject.

**TABLE 5:** ABS applied to the purification of phycobiliproteins.

Species	Tissue disruption/Extraction method	System parameters	Yield/Extraction efficiency/Recovery/PE purity index/Selectivity	Yield/Extraction efficiency/Recovery/PC purity index	References
*Porphyridium cruentum*	Phosphate potassium buffer + Bead mill	Bead mill + isoelectric precipitation + ABS (PEG- potassium phosphate) + ultrafiltration	54%	A_545_/A_280_ = 4.2	Ruiz-Ruiz et al. (2013)
		*V*_r_ = 3.0			
		PEG 1000 g.gmol^−1^			
		TLL 45% (w/w)			
		System pH 7.0			
*Porphyridium cruentum*	—	Polyethylene glycol (PEG)	A_545_/A_280_ = 3.2 92% recovery	—	Benavides and Rito-Palomares (2008)
*Porphyridium cruentum*	Ultrasonic bath	PEG/sulphate + isoelectric precipitation	A_545_/A_280_ = 4.1 72% yield	—	Hernandez-Mireles and Rito-Palomares (2006)
		*V*_r_ = 1.0			
		PEG 1000 g.gmol^−1^			
		System pH 7.0			
*Porphyridium cruentum*	Glass beads	Polyethylene glycol-phosphate	A_545_/A_280_ = 2.8 ± 0.2 82% yield	—	Benavides and Rito-Palomares (2005)
		V_R_ = 1.0			
		PEG 1000 g.gmol^−1^			
		TLL 50%w/w			
		System pH 7.0			
		V_R_ = 0.3	—	A_615_/A_280_ = 2.1 ± 0.2 98% yield	
		PEG 1450 g.gmol^−1^			
		TLL 3%w/w			
		System pH 7.0			
*Porphyridium cruentum*	Glass beads	Polyethylene glycol-phosphate	A_545_/A_280_ = 2.9 77% yield	—	Benavides and Rito-Palomares (2004)
		Vr = 1.0			
		PEG 1450 g.gmol^−1^			
		TLL 24.9% w/w			
		System pH 8.0			
*Anabaena cylindrica*	Sodium phosphate (20 nM, pH 7.0)	Dextran T6 + Copolymer Pluronic PE 6400	—	A_620_/A_280_ = 2.16	Sintra et al. (2021)
*Gracilaria* sp.	Maceration	10 wt% of surfactant and 0 or 0.3 wt% of SAIL	Recovery of PE = 78.8 ± 0.8%	Selectivity = 13.6 ± 0.1	Vicente et al. (2019)
				0.047 ± 0.004	
	Maceration + microfluidics + ultrafiltration	10 wt% of surfactant and 0 or 0.3 wt% of SAIL	416 mg of R-PE/g dry biomass		Seručnik et al. (2020)

The first work done in this context was by Benavides and Rito-Palomares (2004). In this work, the authors studied polyethylene glycol (PEG) with different molecular weights, 1,000, 1,450, 3,350, and 8,000 g. gmol^−1^, obtaining the best purity for PE with PEG 1450, TLL 24.9% (w/w) at a pH of 8.0. Later, they showed that the best purity of PE was obtained for PEG 1000, (TLL 50% w/w and system pH 7.0) and PEG 1450 for PC. Later, Antelo et al. (2007) continued to test conventional ABS. Benavides and Rito-Palomares (2008) found that PEG 1000, beyond the higher yield for PE, induced the change of two conditions of the system: increased volume ratio (1.0 for 4.5) and decreased the TLL (50% for 45%), allowing to achieve a purity of 3.2.

The ABS process can also be used combined with other processes, such as isoelectric precipitation. Hernandez-Mireles and Rito-Palomares (2006) used three processes for PE extraction: cell disruption behind sonification, isoelectric precipitation with the addition of HCl, and PEG/phosphate ABS extraction achieving an excellent purity of 4.1. Ruiz-Ruiz et al. (2013) obtained an excellent purity (4.2) through four steps: cell disruption through bead mill, isoelectric precipitation, ABS, and lastly ultrafiltration. Later, in 2020, Sintra and collaborators (2020) used ABS based on copolymers and dextran to improve the purity and stability of C-PC. The extraction with sodium phosphate produced an extract with a purity of 0.52, and after purification, the purity was increased by 4-fold. Vicente et al. (2019) tested several surfactants to isolate and maintain the R-PE structural integrity, identifying benzyldodecyldimethyl-ammonium bromide as the most adequate. Later, the same author studied the effect of using microfluidic devices to make the intensification of the process of purification of phycoerythrin, achieving very good results as well (Seručnik et al., 2020).

## Phycobiliproteins applications

The study of phycobiliproteins as bioactive compounds has been growing in different areas including cosmetics, food, textile, and pharmaceutical, because they are obtained from renewable abundant sources, have good stability, biocompatibility, and bioactivity (Guedes et al., 2011; Manivasagan et al., 2017). In this section, studies of the application of phycobiliproteins for different purposes will be reviewed and discussed.

### Food applications

The use of synthetic dyes in the food industry is potentially harmful to human health, due to their toxicity. Phycobiliproteins may play a major role as natural food colorants in their water-soluble protein-bound forms. Generally, these pigments present an enhanced solubility as well as high stability in the pH range of 4–10 (Galland-Irmouli et al., 2000; Munier et al., 2014). Phycoerythrin holds potential as an alternative red natural food colorant. Yet more relevant, allophycocyanin and PC present bluish-green and dark blue hues, respectively, which are rarely found in other natural sources. However, to guarantee the stability of the phycobiliproteins in the desired final product, some conditions must be considered, such as temperature, pH, and light (Manirafasha et al., 2016).

The addition of acids, salts, and sucrose was proven to help prevent the denaturation of phycobiliproteins. Mishra et al. (2010) found that among the preservatives studied: citric acid, sucrose, sodium chloride, and calcium chloride, citric acid was the best preservative for C-PE as it acts as a chelator and reduces the pH preventing protein degradation. The stability of PE applied in three carbonated drinks, namely, Lehar soda, 7′UP, and TATA mineral water were evaluated. Although for the 7′UP drink, the color was retained for more than 30 days, for the other drinks it was stable for only 3 days. The authors argue that the sugar present in 7′UP acted as a preservative retaining the colour for longer times (Sudhakar et al., 2014). The study of thermokinetic stability in PC and PE extracted from *Nostoc sp.* in preservatives showed that for both phycobiliproteins, benzoic acid is the best preservative at 4°C compared to citric acid, sucrose, ascorbic acid, and calcium chloride, at 4°C, 25°C, and 40°C (Kannaujiya and Sinha 2016b). Zhang et al. (2020) evaluated the stability of C-PC in whey protein in acidified conditions during light storage. It was found that whey protein helped protect C-PC from color degradation in light. The color stability of PE from a crude extract from Rhodomonas salina was studied, the best conditions being established under white fluorescent light for 8 h, a maximum temperature of 40°C, 20% (v/v) of ethanol, and pH range of 3.9–8. (Marraskuranto et al., 2019).

The impact of the addition of these pigments in dairy products was also evaluated by some authors. PE and PC extracted from Atacama Cyanobacteria had chemical stability at pH 5-8 and temperature up to 50°C. The addition of the pigments to skim milk fortified allowed for higher scores in sensory tests (Galetović et al., 2020). In another study, three types of milk bases were compared: milkshakes, liquid yogurts, and yogurts. Successfully, all the products evaluated exhibited the pink color of B-PE, with proven stability (García et al., 2021). One technique used to improve pigment stability is microencapsulation, which consists of protecting some material from the environment in which it is contained. Ganesan and Shanmugam (2020) encapsulated PE with kappa-carrageenan and guar-gum to enhance the stability and functionality of the pigment in ice cream, resulting in better rheology and augmented intensity of pink color over 90 days of storage.

### Nutraceutical and pharmaceutical applications

Oxidative stress is the imbalance between free radicals and antioxidants in the body. This imbalance can cause various diseases such as diabetes, cancer, and inflammation, just to mention a few. To prevent and treat these diseases, there are already some phytochemicals such as tocopherol, caffeic acid, and zeaxanthin (Pagels et al., 2019). PC and PE have also been studied for this purpose due to their antioxidant, antibacterial, anticancer, and anti-inflammatory activities. The antioxidant activity of PC isolated from *Anabaena* biomass proved to be good against DPPH (2,2-diphenyl-1-picrylhydrazyl) and ABTS (2.2′-azinobis-3-ethylbenzothiazoline-6-sulfonic acid) free radical and was able to attenuate the liver structural deformations caused by carbon tetrachloride (CCL_4_) in rats (Osman et al., 2020). Fernández-Rojas et al. (2015) were the first to report that C-PC prevents mitochondrial dysfunction and increases oxidative defense in mice. This study motivated Wang et al. (2020) to study the effects of PC against doxorubicin (DOX), a chemotherapeutic agent that causes Chemotherapy-Induced Cognitive Impairment (CICI), a common detrimental effect of cancer treatment. Studies in mice have shown that PC has the potential to treat CICI as it improves established DOX-induced cognitive deficits, due to the inhibition of neuroinflammatory and oxidant stress and attenuation of mitochondrial and synaptic dysfunction. In addition to the antioxidant activator, PE is also known to be effective against age-related diseases. In *in vitro* experiments, the antioxidant and immunomodulation potential of C-PC extracted from *Spirulina* were also evaluated, without revealing any toxicity in the mice (Grover et al., 2021). Yoshimoto et al. (2019) also found immunomodulation activity, as well as anti-inflammatory actions in the mucosal immune responses. R-PE can inhibit the growth of subcutaneous transplanted tumors, repair damaged mucosa to protect the intestinal barrier, and regulate the immune function of mice (Qi et al., 2019). Regarding PC, studies have indicated that this pigment can induce apoptosis, one of the important mechanisms in the inhibition of cancer cell proliferation, of multiple non-small cell lung cancer cells and colorectal cancer cells (Hao et al., 2019; Hamdan et al., 2021).

Photodynamic therapy (PDT) is a treatment that combines light and photosensitizing agents to destroy cancer cells. Phycobiliproteins can be used as photosensitizers because they can emit strong fluorescence after being irradiated with a laser (Li et al., 2019). A study on the inhibition of β-site amyloid precursor protein cleaving enzyme-1 (BACE1) by PE revealed potential in the application of C-PE as a therapeutic agent in Alzheimer’s disease (Chaubey et al., 2019). Lian et al. (2020) found that treatment in rats with C-PC attenuated gastric ulcers by suppressing oxidation and inflammation and increasing gastroprotection. β-carotene and PC added to the standard diet of Nile tilapia, allowed us to conclude that the fish with a diet supplemented with PC had a higher survival rate, with an increase in intestinal digestive enzymes such as amylase, trypsin, and lipase, and improved hematological parameters such as immunoglobulin M (IgM), catalase, and total antioxidant capacity (T-AOC) (Hassaan et al., 2021).

### Fluorescence applications: Sensing and solar energy harvesting and conversion

Besides the application of phycobiliproteins in photodynamic therapy, another application is their use as fluorescent probes for analyte sensing. You et al. (2020) developed a luminescent nanoprobe based on the upconversion of nanoparticles conjugated with PC to detect the bioactivity of myeloperoxidase, a protein that causes inflammation-related diseases. Yang et al. (2020) have found a viable method for the detection of ochratoxin A and zearalenone, a quantitative fluorescence image analysis based on multicolor upconversion nanocrystal (UCN)-encoded microspheres. PE was also used for the detection of transcription factors and as a fluorescent label in the microsphere (Sun et al., 2021).

Metals are by-products of several industrial processes that present toxic, corrosive, and malodorous properties. The study of PE as a hydrosulphide selective optical probe has shown promising results in freshwater and effluent samples through the fluorescence ‘turn off’ phenomenon, (Ghosh and Mishra 2020). The same mechanism was applied to mercury, in which C-PE was successfully used as a natural agent for the selective detection of environmentally hazardous Hg^2+^ (Ghosh et al., 2020). R-PE has shown potential when conjugated with silver nanoparticles (AgNPs) for the detection of Cu^2+^. Xu et al. (2019) reported that the addition of the ion Cu^2+^ to R-PE-AgNPs leads to a decrease in fluorescence and color change due to the increasing size of the particle diameter. This change in fluorescence was directly proportional to the concentration of Cu^2+^, therefore this method can be applied to real wastewater samples.

Ghosh et al. (2020b) developed a natural protein-based DNA sensor with PC and graphene oxide, a complex which allowed differentiating DNA from a mixture of other biomolecules (amino acids, sugars, polydispersed exopolysaccharides, other proteins) through ‘turn off, turn on fluorescence. The detailed study of the structure and composition of phycobiliproteins can be an obstacle to their use as proteins from natural sources. Studies have found that the central subunits of PC and PE complexes, although absent from the crystal structures, may be crucial for their stability, and even that PE is the best phycobiliprotein to be used as a fluorescent probe due to the stabilizing effect of its γ subunits (Leney et al., 2017; Kaldmäe et al., 2019).

**FIGURE 1:**
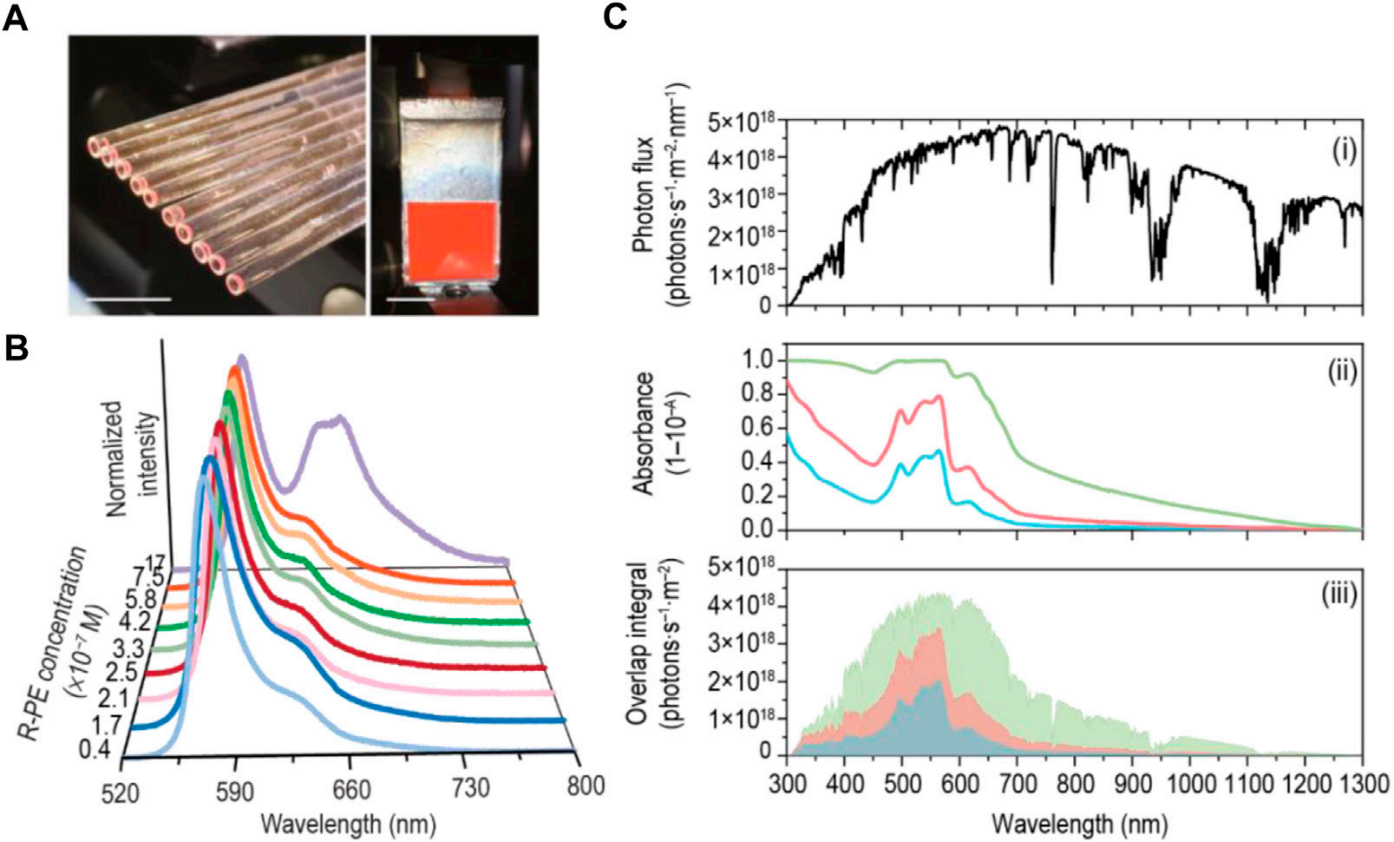
**(A)** Photographs of a bundle of cylindrical LSCs and a planar LSC based on R-PE solutions under AM1.5G. Scale bars: 1 cm; **(B)** Emission spectra of the R-PE solutions excited at 498 nm; **(C)** (i) Solar photon flux on Earth at AM1.5G, (ii) absolute absorbance of 1.7 × 10^–7^ M (blue line), 3.3 × 10^–7^ M (red line), and 17 × 10^–7^ M (green line), and (iii) integral overlap between the solar photon flux and the absolute absorbance. Reproduced with permission from Frias et al., 2019. Copyright 2019, Wiley-VCH Verlag GmbH & Co. KGaA, Weinheim.

Another field where R-PE stands out due to its fluorescence is bio-based luminescent solar concentrators (LSCs, Figure 1A). Frias et al. (2019) used R-PE aqueous solutions to fabricate planar and cylindrical LSCs with maximum optical conversion efficiency values of 6.88%, being the largest among other biomolecules studied such as chlorophyll or Green Fluorescent Protein, Table 6. The high figures of merit arise from the photoluminescence features of the R-PE, namely the emission in the absorption region of typical Si photovoltaic devices (Figure 1B) and high spectral overlap between the R-PE absorption and the sunlight (Figure 1C), which indicated that the most concentrated aqueous solution has the potential to absorb ≈27% of the solar photon flux on the Earth (4.3 × 10^21^ photons∙s^−1^∙m^−2^) (Frias et al., 2019).

**TABLE 6:** Reports of LSCs incorporating phycobiliproteins from algae and comparison of their performance with that of other natural-based molecules.

	Solvent	Fluorophore/Host matrix	Dimensions (cm_3_)	*G*	*η*_*opt*_ (%)	*PCE (%)*	Ref.
Natural molecules	Triton X-100	Phycobilisomes/Acrylamide	2.2 × 2.2×0.05	44	12.5	—	Vossen et al. (2016)
	DCM	Chlorophyll/t-U (5,000)	1.0 × 1.0×0.3	3.3	3.70	0.10	Frias et al. (2018)
	Water	R-PE/Glass container	2.0 × 2.0×1.0	2	6.88	0.27	Frias et al. (2019)
		mScarlet/PDMS slab	2.5 × 2.5×0.6	0.54	2.58	—	Sadeghi et al. (2019)
		eGFP/Glass container	2.0 × 2.0×1.0	2	3.30	0.35	Carlos et al. (2020)
			4.0 × 2.0×1.0	4		0.12	Correia et al. (2022)
		PC/Glass container			2.65	0.21	
Carbon dots		N-CDs/PMMA	2.5 × 1.6×0.1	4.88	4.75	3.94	Li et al. (2017)
		N-CDs/PMMA	2.0 × 2.0×0.2	10	12.2	2.63	Gong et al. (2018)
		N-CDs/PVP	1.8 × 1.8×0.11	4.09	5.02	4.97	Wang et al. (2018)
		N-CDs/custom glass	5 × 2.5×0.42	5.5	4.52	2.49	Mateen et al. (2019)
		N-GQDs/PMMA	2 × 2×0.3	6.7	—	8.77	Saeidi et al. (2020)
		UV-CDs/PVP	10 × 10×0.2	50	1.10	—	Zhou et al. (2018)
		NaOH-CDs/PVP					
		Narrow sized CDs/PVP	10 × 10×nd	4.5	2.70	1.04	Zhao et al. (2021)
			15 × 15×nd	6.8	2.20	1.13	
	Ethanol	CDs/PVP	10 × 10×0.9	2.8	1.60	0.7	Zhao et al. (2019)
	DMF		10 × 10×1	2.5	0.92	—	Zhao (2019)
		OLA-CDs/PLMA	10 × 1.5×0.2	10	1.20	4.65	Zhou et al. (2018)
	Water	N-CDs/PVP	2.5 × 2×0.2	5.5	5.20	4.06	Ma et al. (2019)
	Acetic acid	TPFE-Rho/PMMA	2.5 × 2×0.2	2.8	5.20	4.06	
	Water	b-CDs/PVA	8 × 8×0.8	10	2.30	—	Zdrazil et al. (2020)
	Ethanol	g-CDs/PVP					
		r-CDs/PVP + PEI					
	Methanol	Y-CD/PVP	10 × 10×0.9	2.5	4.3	3.8	Li et al. (2021)
		R-CD/PVP					

t-U(5,000), tri-ureasil organic-inorganic hybrid; PDMS, polydimethylsiloxane; eGFP, enhanced Green Fluorescent Protein; N-CDs, nitrogen-doped CDs; PMMA, polymethylmethacrylate; PVP, polyvinylpyrrolidone; N-GQDs, nitrogen-doped graphene quantum dots; UV-CDs, ultraviolet active carbon-dots; NaOH-CDs, NaOH, treated carbon-dots; OLA, oleylamine; PLMA, poly (lauryl methacrylate); b-CDs, blue-emitting carbon dots; g-CDs, green-emitting carbon dots; r-CDs, red-emitting carbon dots; PEI, polyethylenimine; Y-CDs, yellow carbon dots; R-CDs, red carbon dots.

**FIGURE 2:**
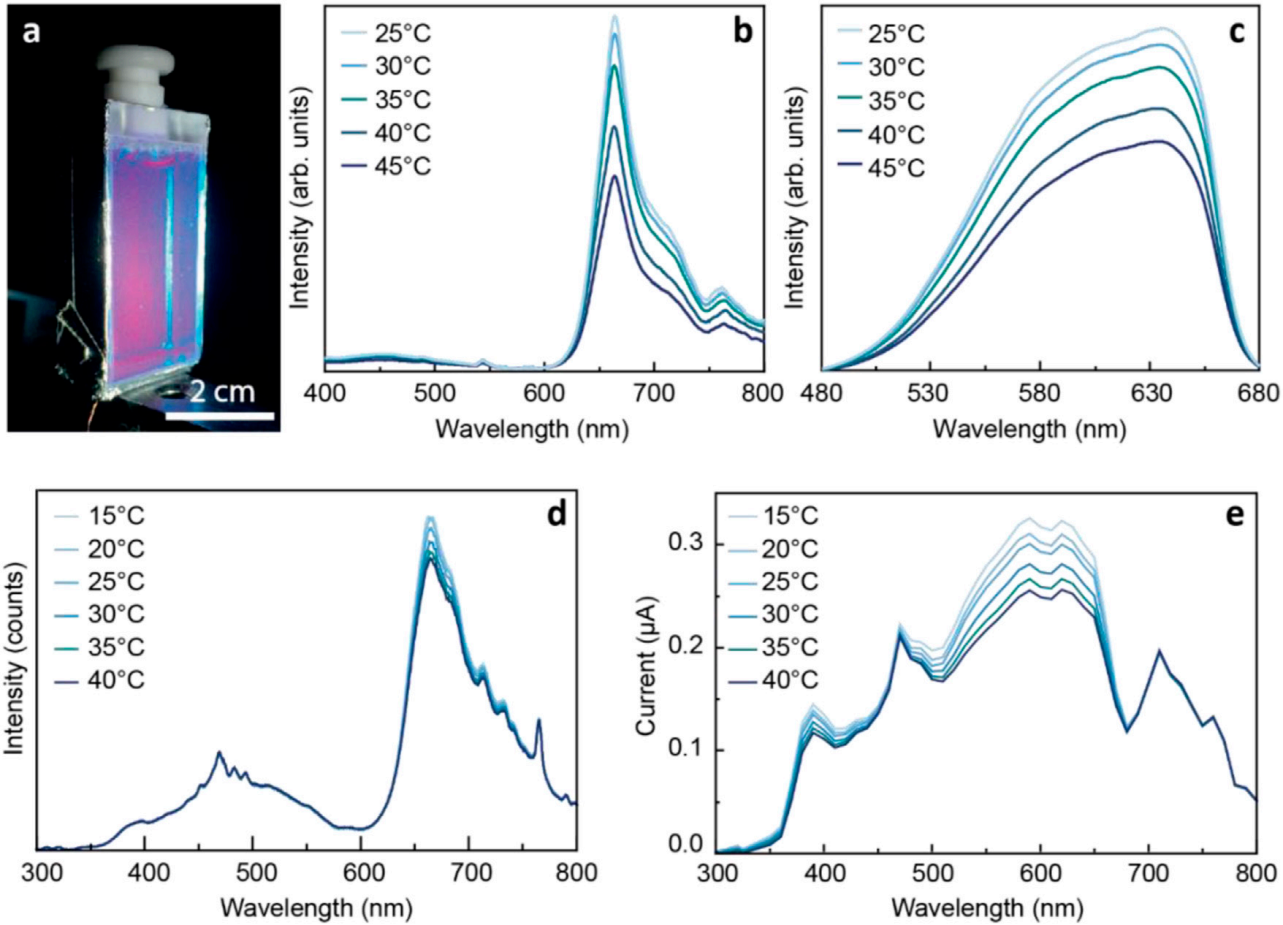
**(A)** Photograph of the LSC/sensor based on a glass container filled with PC-based aqueous solutions under AM1.5G illumination. The PV cell is located at the bottom edge. PC-based optical sensors temperature-dependent **(B)** emission and **(C)** excitation spectra excited at 380 nm and monitored at 715 nm, respectively, and **(D)** emission spectra and **(E)** generated short-circuit current under solar simulator irradiation. Reproduced from (Correia et al., 2022) under a CC BY 4.0 license.

Combining the sensing ability with that of sunlight harvesting, Correia et al. (2022) reported a surprising example of a novel application by fabricating a sustainable solar optical temperature sensor based on PC aqueous solutions (Figure 2A). As PC optical features are temperature-dependent (Figures 2B,C), the electrical output of the PC-based LSC also varies (Figure 2D). After calibration, this device allows us to infer the temperature values from the output voltage of the photovoltaic cell coupled to the LSC. Moreover, the electrical power delivered by the coupled PV cells under solar radiation was enough to power a small circuit able to read voltage values, convert it to temperature and send real-time data through Wi-Fi to a smartphone app or website, bridging these sensors to the Internet of Things (IoT). The goal here was the building integration of photovoltaic and sensing units as smart windows, which could contribute to the future design of zero-energy buildings with enhanced energy consumption management (Correia et al., 2022).

**FIGURE 3:**
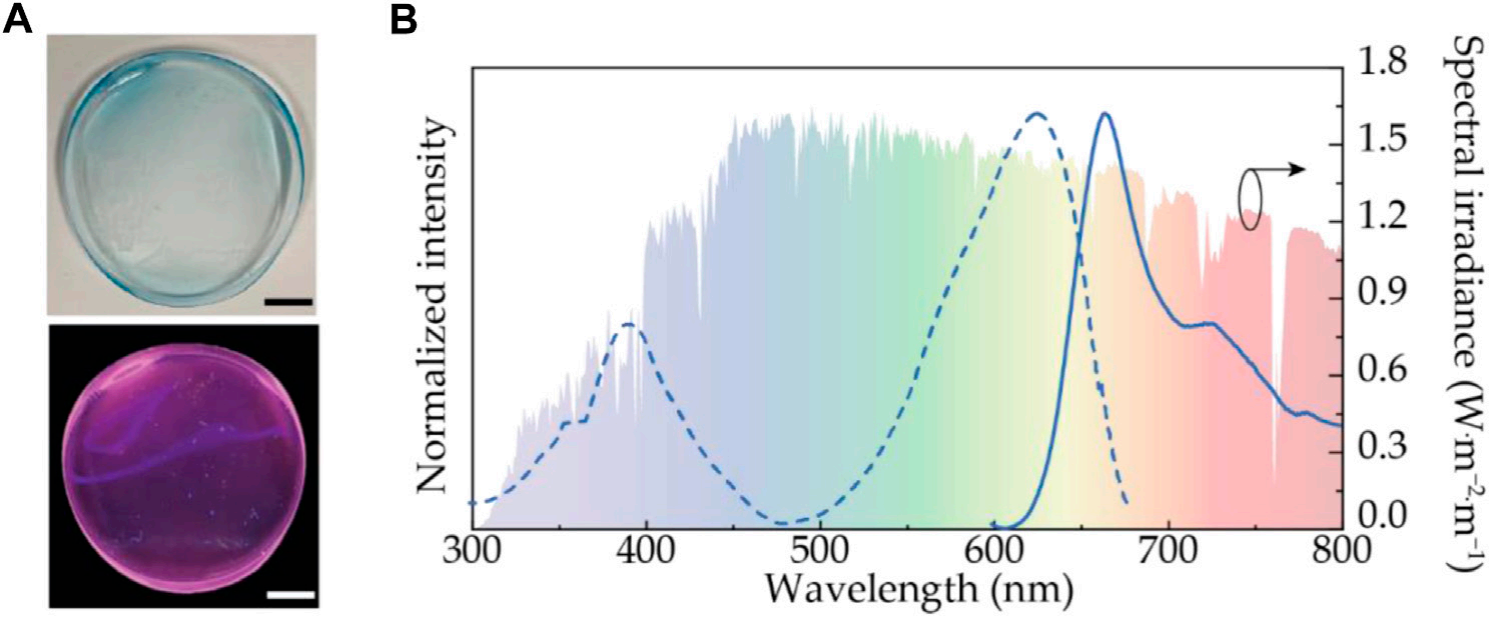
**(A)** Photographs of PC incorporated in PVA (Dias et al., 2022) under white light (top) and UV irradiation at 365 nm (bottom). Scale bars: 10^–2^ m. **(B)** Emission and excitation spectra excited at 575 nm and monitored at 720, respectively. The c-Si spectral response is shown on the right y-axis. **(B)** Excitation spectra for R-PE/PVA, C-PC/PVA, and FX/PVA monitored at 720 nm. The shadowed area represents the AM1.5G solar spectrum (right y-axis). Reproduced from (Dias et al., 2022) under a CC BY 4.0 license.

To improve the processability of the phycobiliproteins, the PC molecules were entrapped into solid matrices, such as poly (vinyl alcohol (PVA) (Dias et al., 2022). It was demonstrated that the ability to down-shift the UV radiation observed for the biomolecules in solution (Figure 2) was kept after their incorporation into the host, Figure 3. Nonetheless, it was observed a decrease in the emission quantum yield (0.09 ± 0.01) when compared to the value found for the aqueous solution together with poor photostability, which suggests molecular aggregation (Zhao et al., 2021). Therefore, this preliminary study reinforces the need for further optimization of the incorporation procedures.

## Conclusion and future perspectives

In this work, the downstream processes reported to recover phycobiliproteins from marine and freshwater biomass were reviewed. The different sources were highlighted (macroalgae, microalgae, and cyanobacteria), and the solvents and techniques used in the extraction and purification of the fluorescent proteins, as well as their main applications taking advantage of being fluorescent/luminescent, were assessed. Most articles reviewed in this work focus only on conventional approaches to extraction and purification, at a laboratory scale paying attention only to the extraction yield and purity level obtained. However, some disadvantages remain, such as poor selectivity, high energetic costs, and high investment in equipment, for example considering the chromatographic techniques (Bleakley and Hayes, 2017). From the publications analysed, it seems that the majority is still using the most conventional solvents, without considering their low selectivity. In this sense, the use of more task-specific solvents is advised. Some authors briefly started to evaluate the effect of some ILs, however, there is a need of choosing the best ones only by their capacity to extract the phycobiliproteins, however without considering too much their economic, environmental impact, safety, or even their potential to develop processes appropriate to scale-up. Taking this into consideration, some other solvents are being used in other fields, but not so much in the marine biorefinery field, although the best results were obtained. Examples of these classes of eco-solvents are the eutectic solvents and more recently, the bio-solvents. Indeed, eutectic solvents were applied in the solid-liquid extraction of proteins as performance boosters (Yue et al., 2021). These are composed of a hydrogen bond acceptor and a hydrogen bond donor and are prepared by mixing natural starting materials with a high melting point, in different molar ratios, to form a liquid. They are simpler to prepare and purify, and of lower costs (Wahlström et al., 2016). Also, they form aqueous biphasic systems (ABS) capable to perform the separation/purification of proteins in a single-step [9], without using chromatography. Bio-solvents, by their turn, are solvents prepared from natural sources, with high biodegradability, abundance, and green credentials, being cyrene the most popular up to now (Sherwood et al., 2014). The set of bio-solvents although small is expanding, and with this expansion more task-specific bio-solvents will be produced on industrial scale, allowing thus to consider them as good alternatives for the development of downstream processes of lower environmental and economic impact, while maintaining or even increasing their capacity as solvents. Nevertheless, aiming at a possible application of some of these processes at an industrial scale much more needs to be defined and investigated, namely the economic and environmental impact of the overall process and stability of the fluorescent proteins. Promising food and pharmaceutical applications of PE were demonstrated primarily at the laboratory scale as pigments and potent antioxidants. Additionally, the photosensitizing and fluorescent properties of these proteins show great potential in varied fields including photodynamic cancer therapy and as organic sunlight harvesters for the improved efficacy of solar panels.

Some works approached the question of the chemical stability of these fluorescent proteins. However, little is known regarding their optical stability. Considering that part of the applications with the highest interest from an economic point of view is related to the optical activity of the phycobiliproteins, the development of strategies to improve the optical stability is a crucial demand, not only in liquid samples but also very important in solid matrices.
